# Matrin3: Disorder and ALS Pathogenesis

**DOI:** 10.3389/fmolb.2021.794646

**Published:** 2022-01-10

**Authors:** Ahmed Salem, Carter J. Wilson, Benjamin S. Rutledge, Allison Dilliott, Sali Farhan, Wing-Yiu Choy, Martin L. Duennwald

**Affiliations:** ^1^ Department of Pathology and Laboratory Medicine, Schulich School of Medicine and Dentistry, Western University, London, ON, Canada; ^2^ Department of Applied Mathematics, Western University, London, ON, Canada; ^3^ Department of Biochemistry, Schulich School of Medicine and Dentistry, Western University, London, ON, Canada; ^4^ Department of Neurology and Neurosurgery, McGill Universty, Montreal, QC, Canada; ^5^ Department of Human Genetics, McGill Universty, Montreal, QC, Canada

**Keywords:** Matrin3, ALS, proteinopathy, intrinsically disordered domains, protein misfolding

## Abstract

Amyotrophic lateral sclerosis (ALS) is a neurodegenerative disorder characterized by the degeneration of both upper and lower motor neurons in the brain and spinal cord. ALS is associated with protein misfolding and inclusion formation involving RNA-binding proteins, including TAR DNA-binding protein (TDP-43) and fused in sarcoma (FUS). The 125-kDa Matrin3 is a highly conserved nuclear DNA/RNA-binding protein that is implicated in many cellular processes, including binding and stabilizing mRNA, regulating mRNA nuclear export, modulating alternative splicing, and managing chromosomal distribution. Mutations in *MATR3*, the gene encoding Matrin3, have been identified as causal in familial ALS (fALS). Matrin3 lacks a prion-like domain that characterizes many other ALS-associated RNA-binding proteins, including TDP-43 and FUS, however, our bioinformatics analyses and preliminary studies document that Matrin3 contains long intrinsically disordered regions that may facilitate promiscuous interactions with many proteins and may contribute to its misfolding. In addition, these disordered regions in Matrin3 undergo numerous post-translational modifications, including phosphorylation, ubiquitination and acetylation that modulate the function and misfolding of the protein. Here we discuss the disordered nature of Matrin3 and review the factors that may promote its misfolding and aggregation, two elements that might explain its role in ALS pathogenesis.

## Introduction

Amyotrophic lateral sclerosis (ALS), also known as Lou Gehrig’s disease in the United States, belongs to a specific category of neurodegenerative diseases known as motor neuron diseases. These diseases are characterized by the degeneration of both the upper motor neurons (Betz cells) in the primary motor cortex, and the lower motor neurons in the anterior horn cells of the spinal cord (van Es et al., 2017; Masrori and Van Damme, 2020). The onset of ALS is age-dependent with the majority of patients diagnosed in the sixth decade of life. ALS affects approximately two in 100,000 individuals, with higher prominence in males than in females (Ingre et al., 2015), and an increased risk for military veterans (Kurtzke and Beebe, 1980; McKee and Robinson, 2014) and athletes (Chen et al., 2007). Typically, ALS leads to death in 2–4 years after onset, killing an estimated 30,000 people worldwide each year (Petrov et al., 2017).

ALS presents clinically with progressive muscle weakness, paralysis, and respiratory failure, which is the major cause of death in patients. ALS can be classified based on its heritability; patients with sporadic ALS (sALS), have no family history of the disease, while patients with familial ALS (fALS) do; sALS accounts for approximately 90% of all cases (Zarei et al., 2015). There is no effective treatment for ALS, with the current standard of care focusing on a multidisciplinary approach to symptom management, employing respiratory and nutritional support, as well as pharamcological interventions (Mejzini et al., 2019). Unfortunatley, riluzole (Rilutek™) (Bensimon et al., 1994; Lacomblez et al., 1996; Andrews et al., 2020) and edaravone (Radicava™/Radicut) (Abe et al., 2017) are the only two FDA-approved medications for treating ALS, and studies show that they can only delay the onset of ventilator-dependence and extend the lifespan of a patient by two to 3 months (Kiernan et al., 2011). Given that oxidative stress (Li et al., 2013; Pollari et al., 2014) and glutamate excitotoxicity (Foran and Trotti, 2009) are two key elements in the biochemistry of ALS, much research has been directed in developing molecules that target these pathways (Petrov et al., 2017; Jaiswal, 2019). In fact, while exact mechanism remains unknown, riluzole and edaravone appear to target elements of glutamate release from motor neurons (Doble, 1996; Jaiswal et al., 2006; Jaiswal, 2017), and ROS production and elimination (Yamamoto et al., 1996; Pérez-González and Galano, 2011), respectively. In any case, most of the attempts to produce novel molecules have been frustratingly unsuccessful (Petrov et al., 2017) and further research needs to identify effective therapeutic targets and strategies for the treatment of ALS, including targeting protein misfolding (Nevone et al., 2020).

ALS and other neurodegenerative disorders, such as Alzheimer’s and Parkinson’s disease, are characterized by defects in protein processing (McAlary et al., 2020) resulting in protein misfolding (Soto, 2003; Parakh and Atkin, 2016; Prasad et al., 2019), mislocalization (Tyzack et al., 2019; Suk and Rousseaux, 2020), and inclusion formation in motor neurons (Soto, 2003; Blokhuis et al., 2013; Jucker and Walker, 2018; Steinacker et al., 2019). Classical neuropathological hallmarks of ALS include ubiquitinated inclusions containing the disordered TDP-43 and FUS (Neumann et al., 2006; Mackenzie et al., 2010; Ikenaka et al., 2020) proteins, although pathology can be heterogeneous with the appearance of other protein aggregates. Notably, recent research into ALS pathogenesis has implicated the partially disordered protein Matrin3 as playing a potentially significant role (Johnson et al., 2014; Malik et al., 2018; Ramesh et al., 2020; Malik and Barmada, 2021). Continued research into Matrin3’s structure and function will be critical to elucidating its interactions with other ALS proteins and nuanced role in the disease. Herein we consider the functional role of both the structured and disordered regions of Matrin3, with particular focus on the potential and documented role of disorder on aggregation and liquid-liquid phase separation (LLPS), as well as the profound effects that both mutation and post-translational modification have on Matrin3 behaviour as it relates to ALS pathogenicity.

## Structure, Disorder, and LLPS in Matrin3

**FIGURE 1:**
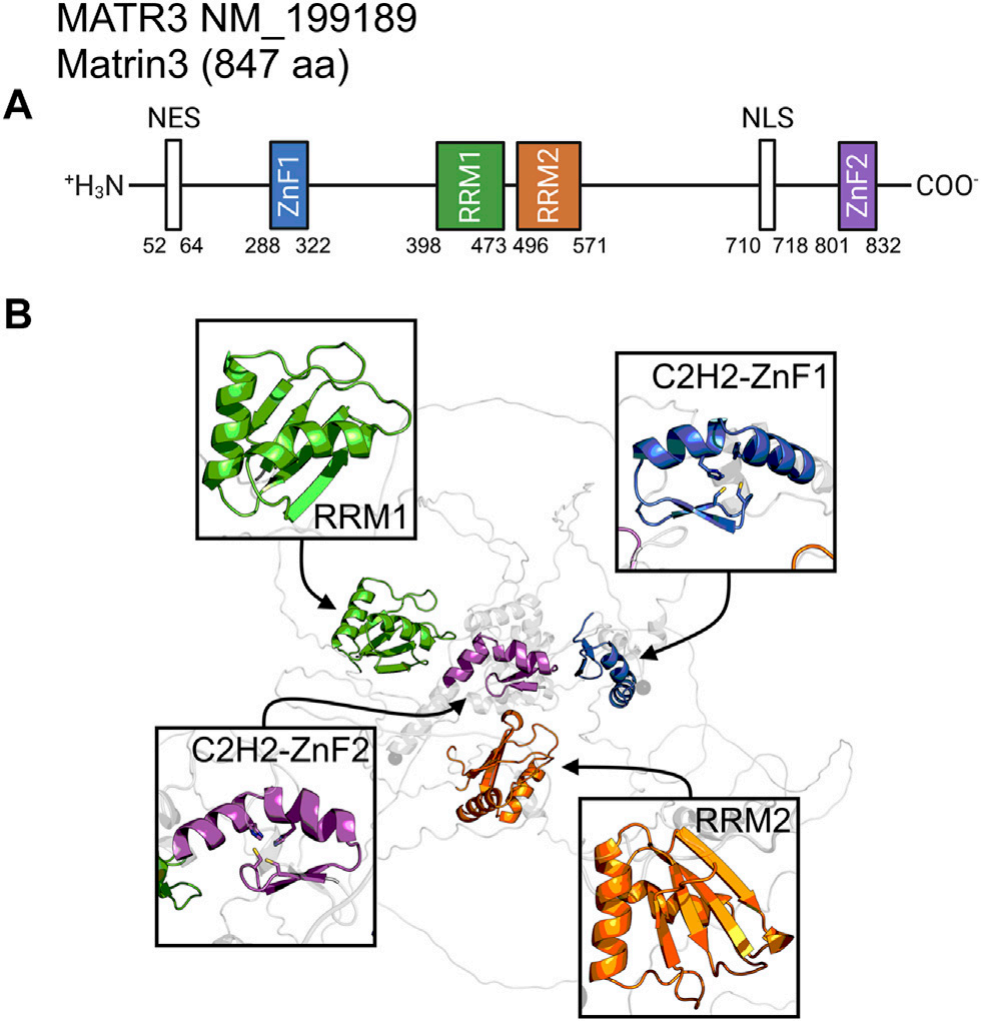
Matrin3 sequence and structure. **(A)** The relative locations of the domains/motifs of Matrin3 (nuclear export signal (NES), Zinc-finger motif (ZnF), RNA-recognition motif (RRM) and nuclear localization signal (NLS)) are indicated approximately. Motif assignment is based on Interpro (Blum et al., 2020) and numerical values indicate the boundaries. **(B)** AlphaFold2 structure prediction of Matrin3, with the two RRM and two ZnF motifs colored to match the sequence graphic. Zinc-coordinating residues are indicated as sticks in the ZnF motif structures.

**FIGURE 2:**
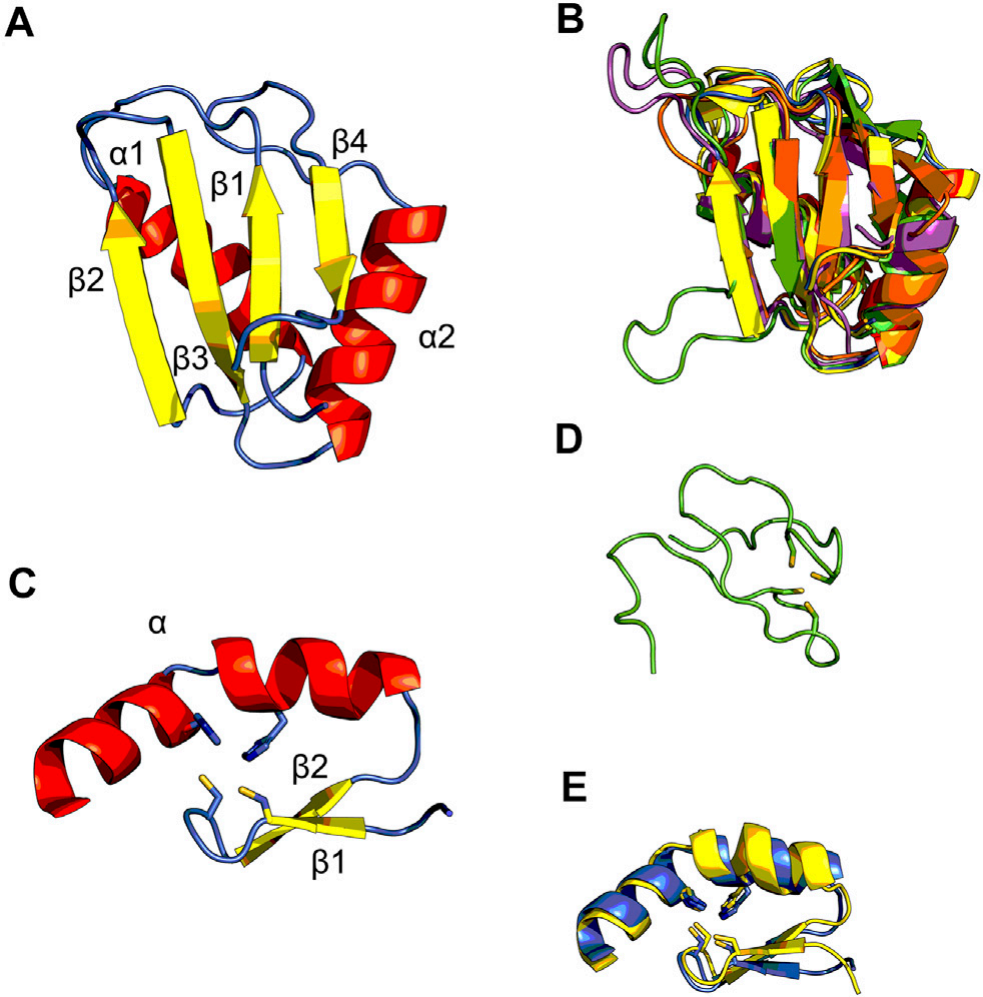
Structural ensembles of Matrin3, FUS and TDP-43. **(A)** RNA-recognition motif (RRM) 1 from Matrin3 and the *βαββαβ*-structure is colored and indicated. **(B)** All RRMs in Matrin3, FUS, and TDP-43 are colored (blue: Matrin3-RRM1, yellow: Matrin3-RRM2, green: FUS, magenta: TDP-43-RRM1 and orange: TDP-43-RRM2) and superimposed. Significant homology is evident and FUS shows the greatest deviation within the ensemble (green). **(C)** C2H2 Zinc-finger motif (ZnF) 1 from Matrin3 and the *ββα*-structure is colored and indicated. Zinc-coordinating residues (two cysteines and two histidines) are indicated as sticks. **(D)** The disordered RanBP2-type ZnF found in FUS is shown. Zinc-coordinating residues (four cysteines) are indicated as sticks. **(E)** Both ZnF motifs in Matrin3 are colored (blue: ZnF1 and yellow: ZnF2) and superimposed, significant homology is evident. All structures are based on AlphaFold2 (Jumper et al., 2021) predictions.

Matrin3 is a 125-kDa nuclear matrix protein encoded by the *MATR3* gene located on chromosome 5 in humans. It is one of the twelve major inner nuclear matrix structural proteins that bind to the nuclear lamina and is ubiquitously expressed in almost all body tissues, with highest expression in brain (Coelho et al., 2016). Computational sequence- and structure-based analysis reveals the protein to be highly disordered (Figure 5, 1A/B), with AlphaFold2 (Jumper et al., 2021) predicting a largely unstructured protein with structure present almost exclusively in its four motifs: two RNA recognition motifs (RRM1 and RRM2) and two zinc-finger motifs (ZnF1 and ZnF2) (Figure 1B). The RRMs are comprised of four beta-strands and two alpha-helices arranged in a *βαββαβ*-fold (Figure 2A). Analysis of the AlphaFold2 structures of Matrin3, FUS and TDP-43 reveal significant structural homology among their RRMs, with FUS showing the largest deviations (Figure 2B). In Matrin3 the RRMs, in particular RRM2, are able to recognize and bind RNA (Salton et al., 2011; Coelho et al., 2016), and this is one of the reasons why Matrin3 has been found to interact with other proteins involved in RNA processing (i.e., TDP-43, FUS and HNRNPA1).

**FIGURE 3:**
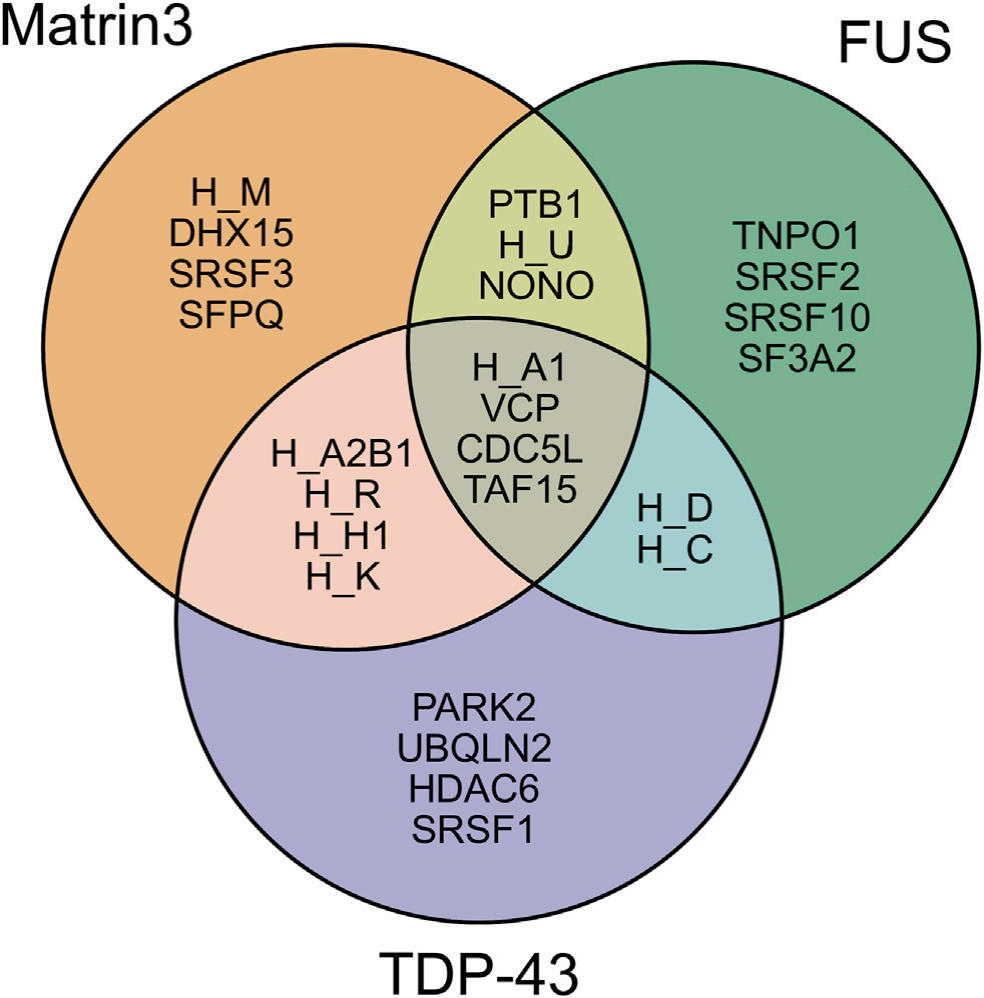
Shared interaction partners between Matrin3, FUS and TDP-43. Venn diagram showing the overlapping protein interaction partners shared between three primary ALS proteins. The appreviation ‘H’ is used for “HNRNP” (i.e., HNRNPA1 = H_A1). Interaction assignment is based on the STRING protein-protein interaction network (Szklarczyk et al., 2018).

Analysis of the association network of Matrin3 reveals many overlapping interactions with TDP-43 and FUS, further pointing to their similar functions in RNA regulation and ALS pathogenesis (Figure 3). The two Cys2His2 (C2H2) Zinc finger (ZnF) motifs in Matrin3 are highly similar (Figure 2E), both adopting a characteristic *ββα*-fold (Figure 2C), and are responsible for binding DNA and have been shown to facilitate protein interactions (Brayer and Segal, 2008; Iradi et al., 2018). The C2H2 ZnF differ from the ZnF found in FUS, namely a disordered RanBP2-type ZnF (Nguyen et al., 2011) that coordinates zinc via four cysteines (Figure 2D). Besides these four motifs, Matrin3 contains a carboxy-terminal nuclear localization signal (NLS) and an amino-terminal nuclear export signal (NES), that function as their names imply.

It is well established that the classical structure-function paradigm whereby an amino acid sequence defines one characteristic, stable, and well-folded protein with a unique biological function does not apply to many proteins, especially in the human proteome. The discovery of a large protein class, comprising both intrinsically disordered proteins (IDPs) that are almost entirely devoid of native structure, and proteins that contain both intrinsically disordered regions (IDRs) and well-structured motifs, has revealed that proteins can readily function as a conformational ensemble (DeForte and Uversky, 2016; Uversky, 2019). These proteins often exhibit a high degree of spatial-temporal heterogeneity and conformational flexibility, with folding free energy landscapes that are relatively flat with a handful of shallow minima (Turoverov et al., 2010; Fisher and Stultz, 2011; Uversky, 2013a). This is a direct consequence of the enrichment of disorder-promoting amino acid residues over and above order-promoting ones within their sequences (Romero et al., 2001; Radivojac et al., 2007; Uversky, 2013b; Theillet et al., 2013). As a result of the flexibility introduced by these residues, IDP/IDRs are capable of interfacing with multiple binding partners via many interactions (Uversky, 2011; Oldfield and Dunker, 2014). While IDP/IDR binding can resemble an induced-fit or conformational selection mechanism (Mollica et al., 2016), IDP/IDRs are interaction specialists (Tompa et al., 2015), that as Uversky summarizes “are promiscuous binders that are never-nude” (Uversky, 2019). IDPs are almost always interacting with their binding partners, forming complexes of various types, including characteristic fuzzy complexes (Tompa and Fuxreiter, 2008; Khan et al., 2013; Sharma et al., 2015), and the strength of these interactions can range from transient and highly specific in signaling networks (Iakoucheva et al., 2002) to ultrahigh affinity interactions between oppositely charged proteins involved in chromatin condensation (Borgia et al., 2018). Particularly relevant to the discussion herein is the disordered nature of proteins implicated in ALS, in particular Matrin3. It is well documented that TDP-43 and FUS, both implicated in ALS, contain significant stretches of intrinsic disorder (Lin et al., 2017; Santamaria et al., 2017; Chiang et al., 2020). Matrin3, a protein that has recently been shown to play a role in ALS pathogenesis, appears to be similarly disordered, which may be related to its pathogenicity.

**FIGURE 4:**
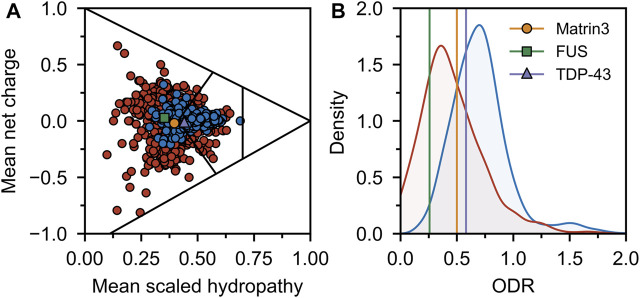
Sequence disorder analysis. **(A)** Charge-hydropathy plot. The plot is based on the method introduced by Uversky *et al.* (Uversky et al., 2000; Uversky, 2017) and compares the mean net charge and mean Kyte-Doolittle hydropathy (Kyte and Doolittle, 1982) (scaled between 0 and 1). Linear boundaries are used to separate various groups of proteins (from left to right extended IDPs, compact soluble proteins, and insoluble proteins). A random sample of ordered (blue) and disordered (red) proteins was taken from the Swiss-Prot (The UniProt Consortium, 2018) and Disprot 7.0 (Piovesan et al., 2016) databases respectively and plotted. Matrin3, FUS and TDP-43 are indicated. **(B)** ODR ratio distribution plot. The same sample as in **(A)** was used to compute the ratio of the order-promoting to disorder-promoting residues with value truncation at 0 and 2. Disordered proteins (red) show a lower ratio (i.e., more disorderpromoting) than ordered proteins (blue).

**FIGURE 5:**
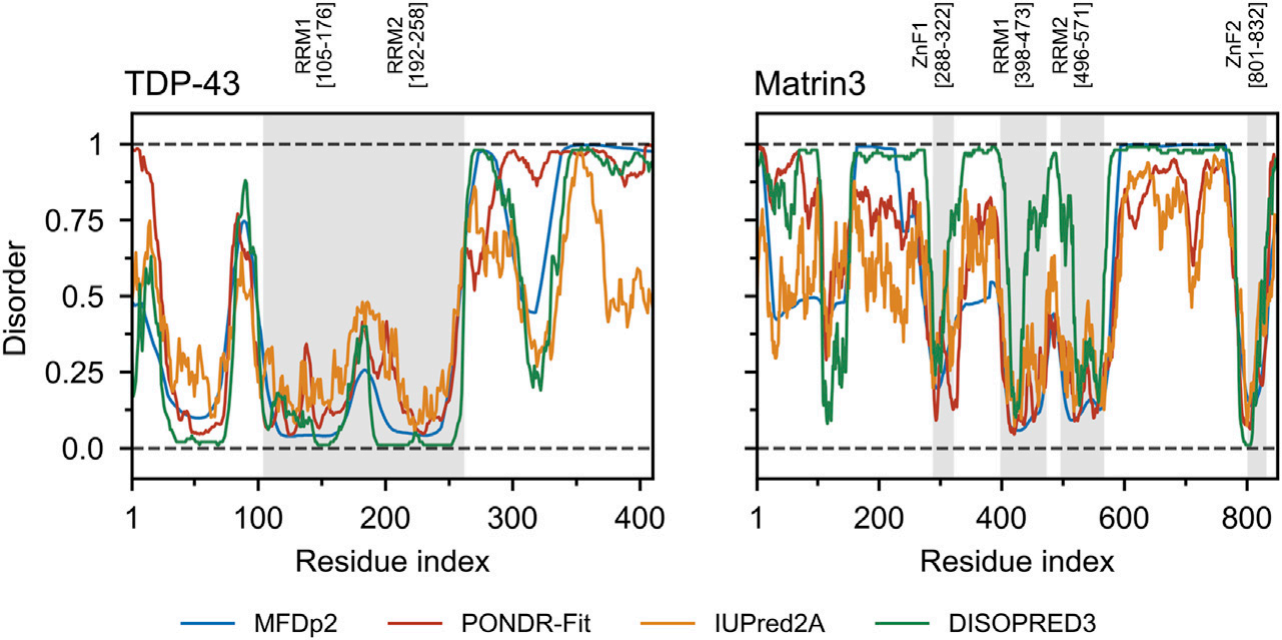
TDP-43 and Matrin3 disorder prediction. Four webservers MFDp2 (Mizianty et al., 2010; Mizianty et al., 2011; Mizianty et al., 2013), PONDR-Fit (Xue et al., 2010), IUPred2A (Mészáros et al., 2018), and DISOPRED3 (Jones and Cozzetto, 2014; Buchan and Jones, 2019) were chosen based on documented performance (Nielsen and Mulder, 2019). A confidence value below 0.5 indicates a residue is likely to belong to an ordered region, while a value above 0.5 indicates a residue is likely to belong to a disordered region. Motifs are indicated and colored in grey as a background on the plot. Motif assignment is based on the InterPro (Blum et al., 2020) and numerical values indicate the boundaries.

Comparing the charge-hydropathy plots (Figure 4A), order-disorder residue (ODR) ratio (Figure 4B) and predicted disorder propensities (Figure 5, Supplementary Table S1) of Matrin3, TDP-43 and FUS reveals that Matrin3 is likely more disordered than TDP-43, but less disordered than FUS. Position-based analysis of Matrin3 disorder reveals that the region between RRM2 and ZnF2 have the highest tendency for disorder, while the region spanning the RRMs has the lowest. Considering the recent computational successes in describing the structural features and dynamics of disordered proteins (Cino et al., 2011; Krzeminski et al., 2012; Do et al., 2014; Ramis et al., 2019; Pietrek et al., 2020; Wilson et al., 2021a; Chang et al., 2021), molecular simulation of specific protein segments could help to more accurately characterize Matrin3, FUS, and TDP-43, by producing a structural ensemble rather than a single frame. Computational methods have also been extended to study protein aggregation propensities (Morriss-Andrews and Shea, 2015), liquid-liquid phase-separation (Paloni et al., 2020; Benayad et al., 2021), IDP-protein interactions (Cino et al., 2013; Do et al., 2016; Karttunen et al., 2018), and the effects of mutation (Vacic et al., 2012; Wilson et al., 2021b) and post-translational modification (Jin and Graeter, 2021; Sieradzan et al., 2021) on protein structure, all potential avenues for investigation of a disordered ALS protein like Matrin3.

It has also been shown that many IDPs can undergo LLPS, and this biophysical phenomenon has emerged as a new paradigm underlying the intracellular assembly of proteins and other macromolecules, including RNA. LLPS is the reversible process of a homogenous fluid de-mixing into two distinct phases, a condensed phase and a dilute phase (Hyman et al., 2014; Alberti and Dormann, 2019; Choi et al., 2020). LLPS has been identified as a critical physical phenomenon underlying the formation of numerous membrane-less cellular organelles, such as P-bodies, stress granules and nucleoli (Kato et al., 2012; Molliex et al., 2015; Nott et al., 2015; Feric et al., 2016; Banani et al., 2017; Boeynaems et al., 2018). In addition to membrane-less organelles, other structures can also be formed through LLPS, including transport channels in the nuclear pore complex (Su et al., 2016), and membrane receptor clusters at the cell membrane (Schmidt and Görlich, 2016).

Defects in LLPS have been speculated to drive protein misfolding and aggregation in different human diseases, including cataracts and neurodegenerative disorders like ALS (Patel et al., 2015; Wegmann et al., 2018; Conicella et al., 2020; Pakravan et al., 2020; Watanabe et al., 2020; Zbinden et al., 2020). For example, proteins inside stress granules can undergo liquid-solid phase transition or can nucleate to form protein aggregates and this process can be accelerated by disease-causing mutations in ALS-associated genes. Specifically, ALS-causing mutations in the gene encoding FUS can result in drastic changes to the biophysical properties of the liquid droplets that form and induces the rapid formation of protein fibrils that are speculated to be cytotoxic (Patel et al., 2015). FUS contains a large intrinsically disordered prion-like domain (PrLD), with an ability to sample many conformational states that likely drives the formation of the liquid state.

These PrLDs, first discovered in yeast are rich in glycine and uncharged amino acids, resulting in a low order and a high degree of flexibility and solvent interaction. Many proteins containing PrLDs have been identified using hidden Markov algorithms (Lancaster et al., 2014; Harrison and Shorter, 2017), and proteomic surveys have revealed PrLDs to be particularly abundant in mammalian RNA-binding proteins (Hennig et al., 2015; Harrison and Shorter, 2017). In many cases, the RNA-binding proteins implicated in ALS (i.e., TDP-43, FUS, etc.) contain PrLDs that mediate their liquid-liquid phase separation. Interestingly, these regions are also where the majority of ALS-causing mutations occur (Gitler and Shorter, 2011; Harrison and Shorter, 2017).

Mutations in the PrLDs result in stark changes to the material properties of droplets formed by these proteins over time and stifle the reversibility of protein aggregate formation (Lin et al., 2015a; Kamps et al., 2021; Saar et al., 2021), leading to the sequestering of additional proteins within the aggregates (Watanabe et al., 2020).

**FIGURE 6:**
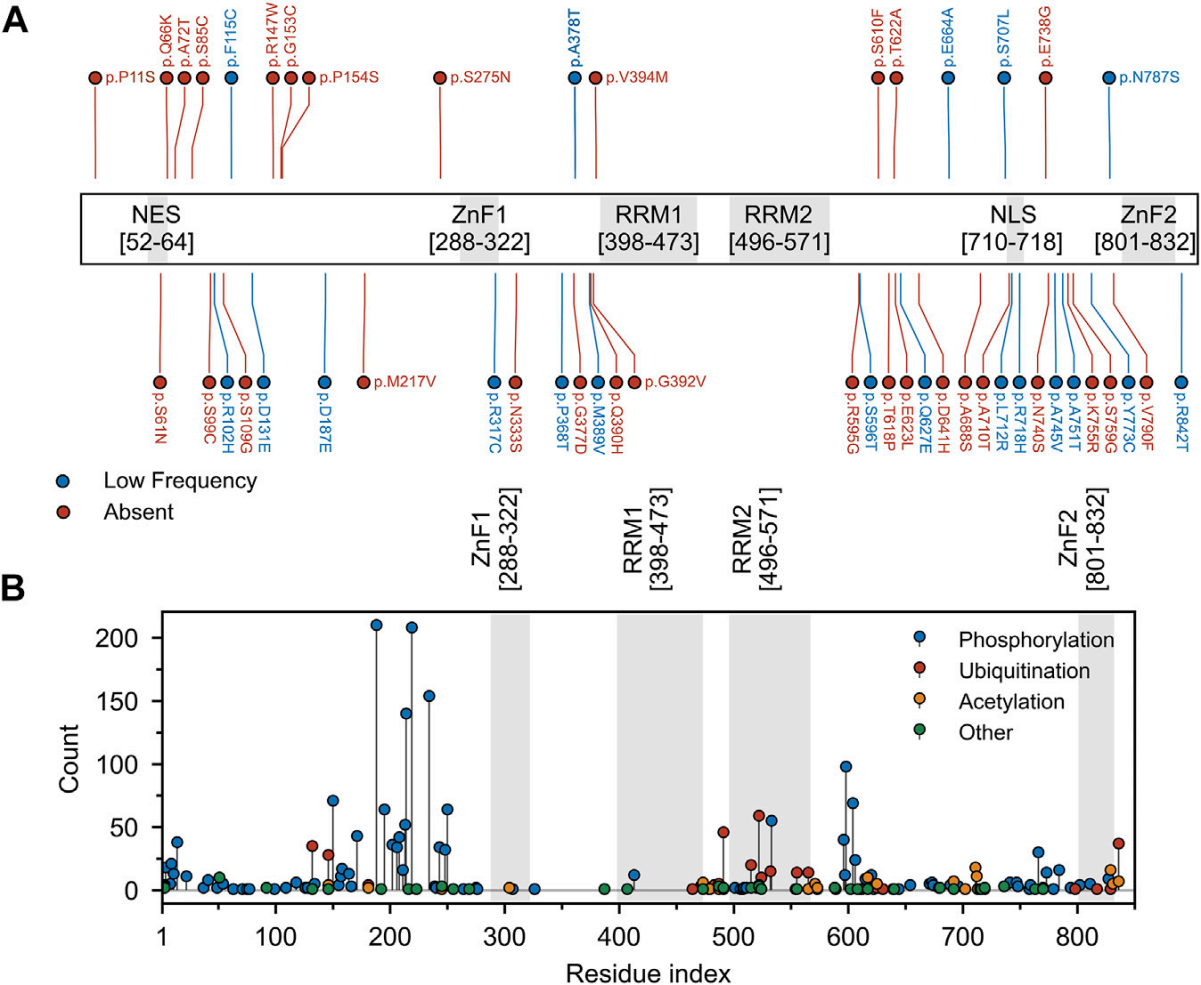
Mutation and post-translational modifications of Matrin3. **(A)** Variant data was plotted as a function of residue index. Variants include those reported to be associated with ALS within the relevant literature and those identified in the ALS Knowledge Portal (Farhan et al., 2019) and/or Project MinE (Project MinE ALS Sequencing Consortium, 2018) ALS whole genomes. Variants observed in the ALS Knowledge Portal or Project MinE ALS whole genomes were only included if the variant had not been observed in the respective study’s control cohort. All previously reported variants were missense variants. Red and blue points indicate variants that were absent or occurred at low frequency (minor allele frequency *<* 0.001) in the gnomAD (v2.1 non-neuro) database. **(B)** Phopshositeplus (Hornbeck et al., 2014) data was plotted as a function of residue index. High- and low throughput studies were simply summed and the height of the most modified residue S188 (390 references/counts) was reduced for plotting purposes. Motifs are indicated and colored in grey as a background on the plot. Motif assignment is based on the InterPro (Blum et al., 2020) and numerical values indicate the boundaries.

By contrast, Matrin3 does not contain any PrLDs, yet it does contain large regions of disorder, and, similar to FUS, almost all of the ALS-associated amino acid substitutions (Figure 6A) and most of the documented post-translational modifications (Figure 6B) occur in Matrin3’s disordered regions. Matrin3 also undergoes liquid-liquid phase separation, and previous work has shown that the N-terminal region (300 amino acids) is required to mediate the phase separation of Matrin3 (Gallego-Iradi et al., 2019). Furthermore, the ALS causing mutation S85C appears to inhibit Matrin3 droplet formation, while P154S and F115C (now no longer considered an ALS-associated Matrin3 variant) do not (Malik et al., 2018; Gallego-Iradi et al., 2019). Furthermore, computational analysis using CamSol and Aggrescan3D, reveals that a significant proportion of the “highly insoluble” and aggregation-promoting residues in Matrin3 are localized to the disordered regions, in particular the N-terminus (Supplementary Figure S1). We therefore speculate that the intrinsically disordered regions of Matrin3 may not only drive its phase-separation behaviour but also promote its ALS-associated misfolding and aggregation.

## Matrin3 Functions and Interactions

As discussed previously, intrinsically disordered proteins are highly flexible, endowing them with the potential to interact with a wide range of binding targets (Uversky, 2019; Oldfield and Dunker, 2014; Uversky, 2011). The regions of IDPs involved in productive binding within a complex are often structurally ill-defined and rarely exist in a single state (Sharma et al., 2015; Khan et al., 2013; Tompa and Fuxreiter, 2008; Freiberger et al., 2021; Arbesú et al., 2018). Although this flexibility may suggest that IDPs form extensive complementarity with their binding partners, IDPs often favor a T-shaped *π*−*π* geometry during protein-protein interactions, implying they deviate from stringent geometric complementarity (Tompa and Fuxreiter, 2008; Espinoza-Fonseca, 2012; Song et al., 2013; Moreira et al., 2013). This lack of complementarity has been linked to function, as it allows for IDPs to interact with many different binding partners. IDPs can interact with their partners through several binding modes, including polymorphic, clamp, flanking and random (Sharma et al., 2015; Tompa and Fuxreiter, 2008). It has been proposed that these alternative modes of binding give rise to the functional promiscuity observed in IDPs often allowing them to act as hubs in protein interactomes (Tompa et al., 2005; Dunker et al., 2005; Haynes et al., 2006; Hu et al., 2017). Directed studies and unbiased proteome- and transcriptome-wide analyses have shown that Matrin3 interacts with DNA, RNA, and a diverse array of proteins, including the ALS-associated RNA-binding proteins TDP-43 (Boehringer et al., 2017) and FUS (Yamaguchi and Takanashi, 2016). Analysis of Matrin3’s association network reveals many overlapping binding partners with both proteins, highlighting the interdependence and connectivity between this trio, and further implicating Matrin3’s role in ALS pathogenesis (Figure 3). We note that Matrin3 is, however, unique among ALS-associated RNA binding proteins, as it possesses two tandem RRMS in addition to two ZnF motifs (i.e., FUS contains only one RRM and one zinc finger, and TDP-43 contains two RRMs and one N-terminal DNA binding motif). This collection of four motifs furnishes Matrin3 with the ability to readily interact with both DNA and RNA. One particular interaction involves the repression of exons by binding to the intronic regions of RNAs with pyrimidine-rich consensus sequences (Uemura et al., 2017). Interestingly, removal of one or both RRMs appears to have no effect on Matrin3 over-expression toxicity, while deletion of ZnF2 is significantly mitigating. This suggests that aberrant overbinding of DNA may be partially responsible for the neurodegenerative effects observed in neurons. This is further supported by observations that deletion of RRM2 is sufficient to promote the formation of phase-separated droplets; a result implying that RNA may prevent or reduce Matrin3 self association (Malik et al., 2018). Here we seek to briefly summarize some of the specific interactions of Matrin3 with RNA and DNA facilitated by its functional domains.

### Binding and Stabilizing mRNA

Matrin3 contains two RRMs, and unsurprisingly has been shown to interact with RNA. Protein-RNA interaction analysis revealed Matrin3 directly interacts with four small noncoding RNAs, and genetic clustering showed 77 genes with reduced expression levels in *MATR3*-depleted cells (Salton et al., 2011). Furthermore, analysis of direct interaction between Matrin3 and mRNAs revealed not only that deletion of RRM2 neutered RNA-association, but that Matrin3 depletion resulted in a decrease in mRNA half-life, suggesting a transcript-stabilizing role for Matrin3 (Salton et al., 2011). Notably, Matrin3 interactions with RNA appear to facilitate associations with other RNA interacting proteins, specifically DHX9, implicated in transport (Tang et al., 1997) and translation of RNAs (Hartman et al., 2006), and HNRNPK, implicated in transcription, splicing, and translation (Moumen et al., 2005). In both cases, association between Matrin3 and these proteins is RNA-dependent.

### Regulation of Alternative Splicing

Alternative splicing of mRNA is a highly regulated and crucial process that enables the expression of multiple proteins from a single mRNA transcript. This is facilitated through the interactions between repressor and enhancer proteins acting on the pre-mRNA (Chen and Manley, 2009). Matrin3 is a strong regulator of splicing events as evidenced by Matrin3 knockdown-dependent dysregulation of 667 alterative splicing events and iCLIP analysis that revealed direct binding of Matrin3 to introns flanking repressed exons (Coelho et al., 2015). Matrin3’s splicing map is consistent with a mechanism whereby initial binding of Matrin3 at specific sites nucleates a cascade of binding across a long RNA region, culminating in repression of the targeted exon. The function of Matrin3 in exon inclusion is less clear. One possibility is that Matrin3 antagonizes the activity of another protein, namely polypyrimidine tract binding (PTB) protein, via interaction with Matrin3’s PTB-RRM interaction motif. PTB functions to repress exons and opposition of PTB function by Matrin3 could counteract this, facilitating inclusion (Coelho et al., 2015).

### Regulation of Chromosomal Distribution

Nuclear matrix proteins (NMP) bind DNA at multiple sites in the genome, and Matrin3 is a highly abundant NMP identified in the inner nuclear matrix (Nakayasu and Berezney, 1991). Minimum-spanning tree (MST) algorithms (Dusser et al., 1987; Dussert et al., 1988; Malyavantham et al., 2008) and 3D fluorescence *in situ* hybridization (FISH) (Langer-Safer et al., 1982) indicated that Matrin3 localizes to extra-nucleolar regions of the nucleus and to relatively distinct sites.

Line profile analysis revealed that Matrin3 was associated with regions in both the gene-poor chromosome 18 and gene-rich chromosome 19 (Zeitz et al., 2009). Analysis using three-dimensional edge detection indicated that Matrin3 was absent from both the perinuclear and perinucleolar heterochromatin and from the inactive copy of the X chromosome but not the active X chromosome, suggesting a possible role in euchromatin organization and function (Zeitz et al., 2009).

### DNA Damage Repair

The DNA damage response is an intricate cellular cascade that is activated in response to DNA damage. One of the most potent activators of the DNA damage response is DNA double-strand breaks (DSBs) (Jackson and Bartek, 2009), and ataxia telangiectasia mutated (ATM) is one of the major nuclear kinases that mediates this response (Khanna et al., 2001). Following the recruitment of ATM to DSB sites, the kinase phosphorylates effector proteins in different response pathways (Marechal and Zou, 2013). Matrin3 is an ATM target as serine 208, located in the disordered N-terminus of Martin3, is phosphorylated following DNA damage (Salton et al., 2010; Niimori-Kita et al., 2018). Matrin3 has also been implicated in the nuclear retention of hyper-edited RNA together with NONO/SFPQ. Matrin3, SFPQ, and NONO may associate with major non-homologous end-joining proteins forming a large hetero-complex and further implicating Matrin3 in DSB response (Bladen et al., 2005; Rajesh et al., 2009; Salton et al., 2010). These results imply that Matrin3 is involved in initiating and carrying out DNA damage response. While the precise mechanism underlying this is not fully understood, it is reasonable to speculate that the disordered nature of Matrin3 facilitates its synergistic interactions with multiple targets in a phospho-dependent manner and might act as a scaffold for the hetero-complex.

## Matrin3 Disease Variants and Pathology

Mutations in the intrinsically disordered regions of protein can drive disease phenotypes by perturbing existing structures (Vacic et al., 2012; Schrag et al., 2021), inducing the formation of novel structural motifs (Bah et al., 2015; Meyer et al., 2018), altering protein-protein interactions (Wong et al., 2020) and promoting self-interaction (Lim et al., 2016), to name a few.

Analysis of two well documented ALS-associated proteins, TDP-43 and FUS, reveals a clustering of disease-producing mutations in their disordered, prion-like domains. Analysis of Matrin3 reveals a similar trend, where previously reported ALS-substitutions are spread across the protein, yet cluster in disordered regions (Figure 6A, Supplementary Table S3). Previous work has documented that mutations in Matrin3 give rise to distinct disease phenotypes, including familial ALS, frontotemporal dementia (FTD), and hereditary distal myopathy (Senderek et al., 2009; Johnson et al., 2014; Millecamps et al., 2014; Lin et al., 2015b; Origone et al., 2015; Leblond et al., 2016; Xu et al., 2016; Marangi et al., 2017). While significant variation is present in the IDRs of Matrin3, only a subset of these variants are documented or predicted to be pathogenic. Why it is that seemingly identical IDR mutations in a protein can give rise to different phenotypes is the focus of much study. Although IDPs lack a fixed 3D structure in their unbound state, when interacting with a binding partner, IDPs can adopt a more structured conformation (Dogan et al., 2014). IDPs can also tolerate mutations in their binding partners that result in increased conformational dynamics, where the IDP adapts to the perturbation in the partner and maintains complementarity at the interface (Huang and Liu, 2013; Mesrouze et al., 2018). The opposite may also be true; increased conformational flexibility, induced by mutation within IDRs, may be readily tolerated and could account for the many non-pathological mutations observed in the IDRs of Matrin3. It is also possible that a single or several mutations could sufficiently perturb the local conformation of a protein, exceeding a flexibility threshold, resulting in a pathological phenotype. The lack of mutations seen in the structured motifs of Matrin3 suggests that, unlike the IDRs, these regions have a much lower “flexibility threshold” and are unable to accommodate most mutations that would severely distort the local conformation of the protein and lead to non-viable phenotypes.

We here note that the frequently reported F115C mutation is no longer believed to be implicated in fALS. While this was one of the first documented mutations alleged to have been causal of fALS (Johnson et al., 2014), recent reanalysis of the kindred studied in the original work revealed that another protein mutation was responsible (Saez-Atienzar et al., 2020). This finding was supported by results observed in an earlier mouse study (Malik et al., 2018). Interestingly, F115C nevertheless results in abundant nuclear inclusions of Matrin3 (Gallego-Iradi et al., 2015). This differs from the S85C mutation, which results in mislocalized Matrin3 that co-aggregates with TDP-43 in the cytosol (Johnson et al., 2014). Notably, even in patients without any disease-producing *MATR3* mutations, mislocalization of Matrin3 has been observed in fALS cases (Dreser et al., 2017) (i.e., patients with fALS caused by *C9orf72* repeat expansion (Johnson et al., 2014)), and in patients with sALS (Tada et al., 2018). Phenotypic variability as a result of mutation is also evident in carriers of the S707L variant, who present with cognitive and behavioral features, suggesting a co-pathology of both ALS and FTD (Marangi et al., 2017). In total there are 49 documented *MATR3* variants observed in patients presenting with both sporadic and familial ALS/FTD (Figure 6, Supplementary Table S2).

Recently, the NIH-funded Clinical Genome Resource (ClinGen) published a standard framework for the evaluation of gene-disease correlations, and efforts are ongoing to classify gene associations across many diseases by Gene Curation Expert Panels (GCEPs), including ALS (Strande et al., 2017). The curation of *MATR3* was recently completed and the gene-disease was given a classification of “Moderate” based on a review of variants previously reported in ALS cases and relevant experimental evidence. This classification indicates that while there is evidence to support a causal role for *MATR3* in ALS and there is no convincing contradictions to the relationship, further evidence is required to attain a “Strong” or “Definitive” classification. In the case of *MATR3*, the ALS GCEP recommends that further functional analyses of potentially pathogenic variants are performed to increase the confidence in the gene-disease relationship.

### Matrin3 Post-translational Modifications

Post-translational modifications, including phosphorylation, ubiquitination and acetylation, readily occur in, and alter, the conformation of disordered proteins (van der Lee et al., 2014; Bah and Forman-Kay, 2016; Wright and Dyson, 2015; Darling and Uversky, 2018; Csizmok and Forman-Kay, 2018; Iakoucheva et al., 2004), increase LLPS potential (Hofweber and Dormann, 2019; Rhoads et al., 2018; Owen and Shewmaker, 2019), and modulate aggregation (Funk et al., 2014; Furukawa et al., 2008; Ercan-Herbst et al., 2019; Schaffert and Carter, 2020). As with the disordered TDP-43 and FUS, post-translational modification of Matrin3 is relatively constrained to its disordered regions with some modifications occurring in RRM2 and ZnF2 (Figure 6B, Supplementary Table S4). Aberrant phosphorylation in TDP-43 has been shown to result in increased cytoplasmic mislocalization and aggregation in neuronal cells (Prasad et al., 2019), and multi-site phosphorylated TDP-43 inclusions in the brain is a hallmark of ALS (Neumann et al., 2007; Hasegawa et al., 2008). Also, FUS phosphorylation tempers its aggregation-prone behaviour and disrupts its ability to undergo phase separation in the presence of RNA (Monahan et al., 2017). Matrin3 is also a phosphoprotein with multiple phosphorylation sites for tyrosine and serine/threonine kinases (Hibino et al., 1998). Three different protein kinases have been identified that phosphorylate Matrin3, ATM kinase, pyruvate kinase M2 (PKM2) and cAMP-dependent protein kinase (PKA). ATM kinase phosphorylates Matrin3 at S208 in response to DNA double-stranded breaks activating DNA damage repair response (Salton et al., 2010); PKM2 modifies the protein at T239, inhibiting degradation by preventing K48-linked ubiquitination (Kumari and Das, 2019); and PKA phosphorylates S188 in response to NMDA receptor activation and its inhibition prevents phosphorylation and degradation of Matrin3 and neuronal death (Giordano et al., 2005).

Ubiquitin is a highly conserved 76 amino acid protein that is expressed in all eukaryotic cells and can be enzymatically linked to lysine residues of target proteins (Pickart, 2001). Pathological protein inclusions are frequently ubiquitin-positive. Indeed, both TDP-43 and FUS have been found as components in ubiquitin-positive inclusions, characteristic of FTD and ALS (Arai et al., 2006; Neumann et al., 2007; Farrawell et al., 2015). The role of ubiquitination in Matrin3 mislocalization and accumulation in ALS remains to be determined.

Lysine acetylation is another major post-translational modification that regulates the functions of many proteins (Ali et al., 2018). Large-scale proteomic studies have identified ∼ 1750 proteins as substrates for lysine acetylation, including RNA-binding proteins involved in ALS (Cohen et al., 2015; Arenas et al., 2020). Specifically, acetylation of the RRMs in TDP-43, can impair RNA binding that results in the accumulation of insoluble, hyper-phosphorylated TDP-43 species; these have pathological similarities to those observed in FTD (Cohen et al., 2015). Furthermore, FUS has recently been shown to be acetylated in both its nuclear localization sequence and RRM resulting in mislocalization and decreased aggregation, respectively (Arenas et al., 2020). Matrin3 contains many predicted lysine acetylation sites positioned throughout its structured and disordered regions, suggesting that acetylation may play a role in ALS pathogenesis.

In sum, it is evident that post-translational modifications can modulate LLPS and protein aggregation of the ALS proteins TDP-43 and FUS, yet their exact role in Matrin3 pathology remains to be clarified.

## Conclusion

Herein, we summarize findings documenting the role that Matrin3 plays in both fALS and sALS in the context of its intrinsically disordered regions, mutations, and post-translational modifications. We discuss how these disordered regions may contribute to its misfolding, resulting in pathological accumulation and mislocalization and ultimately contributing to ALS pathogenesis. Evidently, there are many important questions that future research will need to address to fully decipher the role of Matrin3 in ALS. Does Matrin3 misfolding contribute to ALS by a gain of toxic function, or a loss of function mechanisms, or a mixture of both? What triggers the intrinsically disordered domains of Matrin3 to misfold and how is Matrin3 misfolding typically prevented by cellular protein quality control? How do the pathological variants of Matrin3 contribute to misfolding, and what protein interactions may they alter? And how do the interactions with other ALS-associated proteins, such as TDP43 and FUS, and many other proteins, alter Matrin3 misfolding and mislocalization? As discussed previously, Matrin3 lacks the PrLDs present in TDP-43 and FUS which mediates their liquid-liquid phase separation. Therefore, it is important to determine what separates Matrin3 from the majority of other IDPs in the human proteome that are not linked with pathological misfolding. Detailed structural investigations, computational analyses, and new effective cellular and animal models together, will help to advance our understanding of the role of Matrin3 in ALS pathogenesis and, more generally, will elucidate how intrinsically disordered proteins and intrinsically disordered regions contribute to protein misfolding in neurodegenerative diseases.
