# Supplementary material for: Matrin3: Disorder and ALS Pathogenesis
Source: Front Mol Biosci. 2022 Jan 10;8:794646. doi: 10.3389/fmolb.2021.794646 (PMC8784776; doi:10.3389/fmolb.2021.794646)
Supplement: Supplementary file 1 [file DataSheet1.PDF]

Supplementary Information: Matrin3: Disorder and ALS Pathogenesis

Ahmed Salem,[†,⊥] Carter J. Wilson,[‡,⊥] Benjamin S. Rutledge,[¶] Allison Dilliott,[§] Sali Farhan,[§,‖] Wing-Yiu Choy,[¶] and Martin L. Duennwald[*,†]

†*Schulich School of Medicine and Dentistry, Department of Pathology and Laboratory Medicine, Western University, London, ON, Canada*

‡*Department of Applied Mathematics, Western University, London, ON, Canada*

¶*Schulich School of Medicine and Dentistry, Department of Biochemistry, Western University, London, ON, Canada*

§*Department of Neurology and Neurosurgery, McGill Universty, Montreal, QC, Canada*

‖*Department of Human Genetics, McGill Universty, Montreal, QC, Canada*

⊥*These authors contributed equally to this work.*

E-mail: martin.duennwald@schulich.uwo.ca

Supplemental Figure

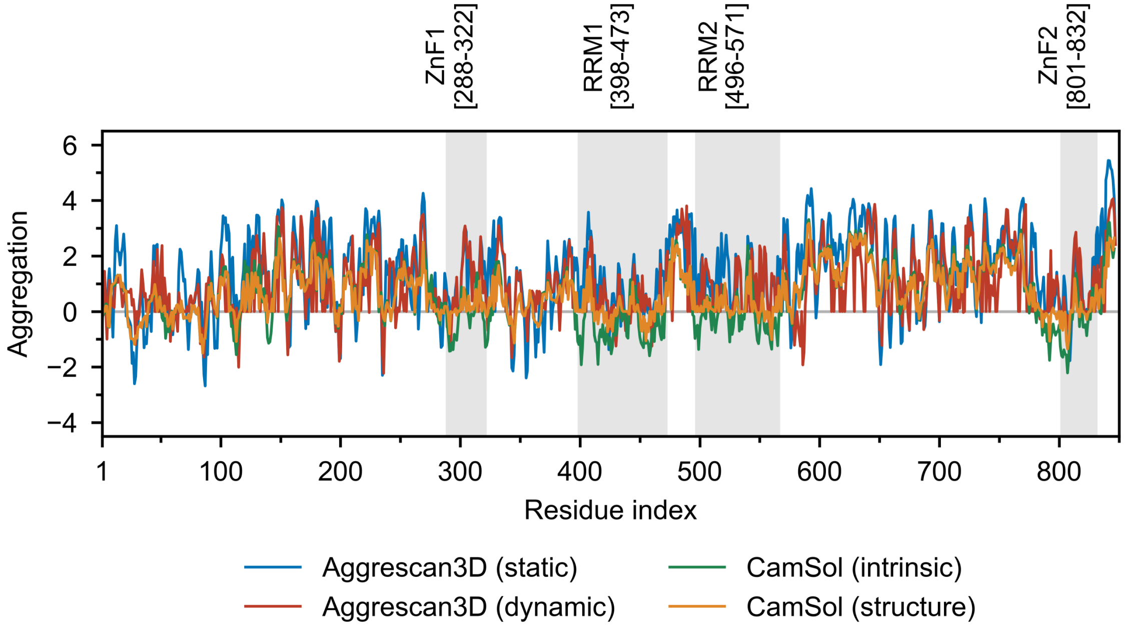

Figure S1: Aggregation propensity of Matrin3. Two webservers CamSol[1,2] and Aggrescan3D[3,4] (A3D) were used to assign an aggregation 'score' at each residue, reflecting its predicted propensity to aggregate (negative values are more aggregation prone, positive values are less). Both webservers were run with two variations: for CamSol, both the intrinsic, and structurally corrected versions (hydrophobic core of a folded domain is not predicted to aggregate) using the AlphaFold2 predicted structure of Matrin3 were run; with Aggresscan3D, the predictor was run in both static and dynamic mode (where a simulation using CABS-Flex[5,6] is first run to characterize the protein dynamics), again using the AlphaFold2 predicted structure of Matrin3. The A3D score was inverted (negative values become positive) to match CamSol.

Supplemental Tables

Table S1: Average disorder propensity. Here we take the residue-wise disorder propensity and compute the mean across the entire protein. The averages across four webservers (MFDp2[7–9], PONDR-Fit[10], IUPred2A[11], and DISOPRED3[12,13]) are: Matrin3 (0.618), FUS (0.835) and TDP-43 (0.436).

	MFDp2	PONDR-Fit	IUPred2A	DISOPRED3
Matrin3	0.573	0.593	0.544	0.763
FUS	0.968	0.833	0.759	0.781
TDP-43	0.441	0.501	0.408	0.393

cDNA	Protein	Cases (N)	gnomAD	CADD	ClinVar	Reference
c.31C>T	p.P11S	1 (107)	NA	18.21	NA	[14]
c.182G>A	p.S61N	1 (6198)	NA	23.2	NA	[15]
c.196C>A	p.Q66K	1 (372)	NA	23.8	NA	[16]
c.214G>A	p.A72T	1 (207)	NA	21.9	NA	[17]
c.254C>G	p.S85C	1 (204)	NA	26	P	[18]
c.296C>G	p.S99C	1 (6198)	NA	25.9	NA	[15]
c.305G>A	p.R102H	1 (3864)	9.61E-06	25.2	VUS	ALS Knowledge Portal
c.325A>G	p.S109G	1 (3864)	NA	23.4	NA	ALS Knowledge Portal
c.344T>G	p.F115C*	NA	8.72E-06	28.8	P	[18]
c.393C>A	p.D131E	1 (6198)	4.81E-06	17.14	VUS	[15]
c.439A>T	p.R147W	1 (200)	NA	30	NA	[19]
c.457G>T	p.G153C	2 (372)	NA	26.7	NA	[16]
c.460C>T	p.P154S	NA	NA	20.6	P	[18]
c.561T>G	p.D187E	1 (6198)	3.36E-05	22.1	VUS	[15]
c.649A>G	p.M217V	1 (3864)	NA	21.9	NA	ALS Knowledge Portal
c.824G>A	p.S275N	1 (107)	NA	17.72	NA	[14]
c.949C>T	p.R317C	1 (6198)	4.81E-06	33	NA	[15]
c.998A>G	p.N333S	1 (6198)	NA	17.01	NA	[15]
c.1102C>A	p.P368T	1 (3864)	9.61E-06	23.6	NA	ALS Knowledge Portal
c.1130G>A	p.G377D	1 (3864)	NA	23.8	NA	ALS Knowledge Portal
c.1132G>A	p.A378T	1 (87)	1.35E-04	22.7	C	[20]
c.1165A>G	p.M389V	1 (3864)	4.81E-06	21.5	NA	ALS Knowledge Portal
c.1170G>C	p.Q390H	1 (3864)	NA	15.82	NA	ALS Knowledge Portal
c.1175G>A	p.G392V	1 (6198)	NA	24.4	NA	[15]
c.1180G>A	p.V394M	1 (247)	NA	21.9	NA	[21]
c.1783A>G	p.R595G	1 (3864)	NA	22.1	NA	ALS Knowledge Portal
c.1786T>A	p.S596T	1 (6198)	4.82E-06	17.2	NA	[15]
c.1829C>T	p.S610F	1 (509)	NA	26.9	NA	[22]
c.1852A>C	p.T618P	1 (3864)	NA	19.78	NA	ALS Knowledge Portal
c.1864A>G	p.T622A	1 (204)	NA	0.173	P	[18]
c.1867G>A	p.E623L	1 (6198)	NA	22.3	NA	[15]
c.1879C>G	p.Q627E	1 (6198)	2.62E-05	17.48	LB	[15]
c.1921G>C	p.D641H	1 (6198)	NA	22.6	NA	[15]
c.1991A>C	p.E664A	1 (372)	2.79E-04	25.1	LB	[16]
c.2062G>T	p.A688S	1 (6198)	NA	5.396	NA	[15]
c.2120C>T	p.S707L	1 (372)	9.65E-06	23.3	NA	[16]
c.2128G>A	p.A710T	1 (3864)	NA	17.9	VUS	ALS Knowledge Portal
c.2135A>G	p.L712R	1 (6198)	3.50E-05	22.6	NA	[15]
c.2149-140G>A	p.R718H	1 (3864)	7.42E-06	17.49	NA	ALS Knowledge Portal
c.2213A>G	p.E738G	1 (149)	NA	24.5	VUS	[23]
c.2219A>G	p.N740S	1 (6198)	NA	18.2	NA	[15]
c.2234C>T	p.A745V	4 (6198)	8.72E-05	21.8	VUS	[15]
c.2251G>A	p.A751T	1 (6198)	2.18E-05	20.9	NA	[15]
c.2149-29A>G	p.K755R	1 (3864)	NA	16.79	NA	ALS Knowledge Portal
c.2275A>G	p.S759G	1 (6198)	NA	22.5	NA	[15]
c.2318A>G	p.Y773C	1 (3864)	5.23E-05	21.7	C	ALS Knowledge Portal
c.2360A>G	p.N787S	1 (372)	3.88E-04	22.1	LB	[16]
c.2368G>T	p.V790F	1 (3864)	NA	26.7	NA	ALS Knowledge Portal
c.2525G>C	p.R842T	1 (3864)	8.19E-05	23.5	VUS	ALS Knowledge Portal

Table S2: (Previous page.) Documented *MATR3* variants. Tabulated variants taken from individual studies and ALS databases, various scores and assignments are indicated. '*' denotes a variant that upon re-analysis was not found to segregate with the ALS phenotype and, therefore, is no longer considered pathogenic for ALS. For variants identified in family studies of ALS, only one member per family carrying the variant was considered in the number of ALS cases. Variants observed in the ALS Knowledge Portal or Project MinE were only included if the variant had not been observed in the respective study's control cohort. Abbreviations: gnomAD, gnomAD (non-neuro v2.1.1) MAF scores; CADD v1.5 *in silico* prediction; CADD, combined annotation-dependent depletion; LB, likely benign; MAF, minor allele frequency; N, total cohort size; NA, not applicable (i.e. variant not observed in database); P, pathogenic; VUS, variant of uncertain significance; C, conflicting interpretations of pathogenicity.

Table S3: Documented Matrin3 functions and corresponding studies.

Predicted Function of MATR3	Reference
Activation of innate immune response	24
Ca^2/calmodulin (CaM)-binding protein	25
Cell growth and proliferation	26
Co-regulator of Rev in HIV-1 biogenesis	27<br>28<br>29
DNA damage response	27<br>30
Maintenance of neural stem cells	31
Mediating microtubule polymerization	32
Mediation of phase separation into intranuclear droplet structures recruiting TDP43	33
Stabilizing mRNA	27<br>34
Negative regulator of the ZAP-mediated restriction of retroviruses	35
RNA binding and processing	34<br>36<br>37
Regulation of alternative splicing	38<br>27
Regulation of mRNA nuclear export	39
Retention of hyper-edited RNA and dsRNA	40<br>41
Activation of N-methyl-D-aspartate receptors and induction of DNA double-strand breaks	27
Transcription activator	27<br>34

Table S4: Post-translational modification list. Abbreviations are used accordingly: phosphorylation: P, acetylatyion: A, SUMOylation: S, and ubiquitination: U. Data was collected from PhosphoSitePlus[42].

Residue	PTM	Residue	PTM	Residue	PTM
S2	A	K304	A	T622	P
K3	A	K391	A	K623	U
S4	P	K464	U	K630	S, U
S9	P	K473	U	S654	P
S11	P	K478	S, U	S671	P
S14	P	K479	U	S673	P
S22	P	K483	U	S674	P
S41	P	K487	S	T679	P
S118	P	K491	S, U	S689	P
S126	P	S509	P	K702	U
K132	S, U	S511	P	K719	S
K146	S, U	K515	S, U	K736	S
T150	P	K522	A, U	T741	P
S157	P	K524	U	S747	P
Y158	P	K532	U	S759	P
S164	P	S533	P	S766	P
K181	U	K554	S, U	K770	S
S188	P	K555	S, U	K798	U
S195	P	K565	U	K817	U
Y202	P	K571	A, U	K829	U
S206	P	K573	U	K836	S, A, U
S208	P	K589	U		
S211	P	S596	P		
Y219	P	S598	P		
S234	P	S604	P		
K245	S	S606	P		
S264	P	S610	P		
K269	S	K611	U		
S275	P	K617	S		

References

(1) Sormanni, P.; Aprile, F. A.; Vendruscolo, M. The CamSol Method of Rational Design of Protein Mutants with Enhanced Solubility. *J. Mol. Biol.* **2015**, *427*, 478–490.

(2) Sormanni, P.; Amery, L.; Ekizoglou, S.; Vendruscolo, M.; Popovic, B. Rapid and accurate in silico solubility screening of a monoclonal antibody library. *Sci. Rep.* **2017**, *7*.

(3) Conchillo-Solé, O.; de Groot, N. S.; Avilés, F. X.; Vendrell, J.; Daura, X.; Ventura, S. AGGRESCAN: a server for the prediction and evaluation of "hot spots" of aggregation in polypeptides. *BMC Bioinform.* **2007**, *8*.

(4) Kuriata, A.; Iglesias, V.; Pujols, J.; Kurcinski, M.; Kmiecik, S.; Ventura, S. Aggrescan3D (A3D) 2.0: prediction and engineering of protein solubility. *Nucleic Acids Res.* **2019**, *47*, W300–W307.

(5) Kuriata, A.; Gierut, A. M.; Oleniecki, T.; Ciemny, M. P.; Kolinski, A.; Kurcinski, M.; Kmiecik, S. CABS-flex 2.0: a web server for fast simulations of flexibility of protein structures. *Nucleic Acids Res.* **2018**, *46*, W338–W343.

(6) Kurcinski, M.; Oleniecki, T.; Ciemny, M. P.; Kuriata, A.; Kolinski, A.; Kmiecik, S. CABS-flex standalone: a simulation environment for fast modeling of protein flexibility. *Bioinformatics* **2018**, *35*, 694–695.

(7) Mizianty, M. J.; Stach, W.; Chen, K.; Kedarisetti, K. D.; Disfani, F. M.; Kurgan, L. Improved sequence-based prediction of disordered regions with multilayer fusion of multiple information sources. *Bioinformatics* **2010**, *26*, i489–i496.

(8) Mizianty, M. J.; Zhang, T.; Xue, B.; Zhou, Y.; Dunker, K. A.; Disfani, V. N., Uversky; Kurgan, L. In-silico prediction of disorder content using hybrid sequence representation. *BMC Bioinformatics* **2011**, *12*.

(9) Mizianty, M. J.; Peng, Z.; Kurgan, L. MFDp2. *Intrinsically Disord. Proteins* **2013**, *1*, e24428.

(10) Xue, B.; Dunbrack, R. L.; Williams, R. W.; Dunker, A. K.; Uversky, V. N. PONDR-FIT: A meta-predictor of intrinsically disordered amino acids. *Biochim. Biophys. Acta Proteins Proteom.* **2010**, *1804*, 996–1010.

(11) Mészáros, B.; Erdős, G.; Dosztányi, Z. IUPred2A: context-dependent prediction of protein disorder as a function of redox state and protein binding. *Nucleic Acids Res.* **2018**, *46*, W329–W337.

(12) Buchan, D. W. A.; Jones, D. T. The PSIPRED Protein Analysis Workbench: 20 years on. *Nucleic Acids Res.* **2019**, *47*, W402–W407.

(13) Jones, D. T.; Cozzetto, D. DISOPRED3: precise disordered region predictions with annotated protein-binding activity. *Bioinformatics* **2014**, *31*, 857–863.

(14) Tripolszki, K.; Gampawar, P.; Schmidt, H.; Nagy, Z. F.; Nagy, D.; Klivényi, P.; Engelhardt, J. I.; Széll, M. Comprehensive Genetic Analysis of a Hungarian Amyotrophic Lateral Sclerosis Cohort. *Front. Genet.* **2019**, *10*, 732.

(15) Consortium, T. H. R. A reference panel of 64,976 haplotypes for genotype imputation. *Nat. Genet.* **2016**, *48*, 1279–1283–668.

(16) Marangi, G.; Lattante, S.; Doronzio, P. N.; Conte, A.; Tasca, G.; Monforte, M.; Patanella, A. K.; Bisogni, G.; Meleo, E.; La Spada, S.; Zollino, M.; Sabatelli, M. Matrin 3 variants are frequent in Italian ALS patients. *Neurobiol. Aging* **2017**, *49*, 218.e1–218.e7.

(17) Lin, K.-P.; Tsai, P.-C.; Liao, Y.-C.; Chen, W.-T.; Tsai, C.-P.; Soong, B.-W.; Lee, Y.-C. Mutational analysis of MATR3 in Taiwanese patients with amyotrophic lateral sclerosis. *Neurobiol. Aging* **2015**, *36*, 2005.e1–2005.e4.

(18) Johnson, J. O.; Pioro, E. P.; Boehringer, A.; Chia, R.; Feit, H.; Renton, A. E.; Pliner, H. A.; Abramzon, Y.; Marangi, G.; Winborn, B. J.; Gibbs, J. R.; Nalls, M. A.; Morgan, S.; Shoai, M.; Hardy, J.; Pittman, A.; Orrell, R. W.; Malaspina, A.; Sidle, K. C.; Fratta, P.; Harms, M. B.; Baloh, R. H.; Pestronk, A.; Weihl, C. C.; Rogaeva, E.; Zinman, L.; Drory, V. E.; Borghero, G.; Mora, G.; Calvo, A.; Rothstein, J. D.; ITALSGEN,; Drepper, C.; Sendtner, M.; Singleton, A. B.; Taylor, J. P.; Cookson, M. R.; Restagno, G.; Sabatelli, M.; Bowser, R.; Chió, A.; Traynor, B. J. Mutations in the Matrin 3 gene cause familial amyotrophic lateral sclerosis. *Nat. Neurosci.* **2014**, *17*, 664–666.

(19) Origone, P.; Verdiani, S.; Poggio, M. B. D.; Zuccarino, R.; Vignolo, M.; Caponnetto, C.; Mandich, P. A novel Arg147Trp MATR3 missense mutation in a slowly progressive ALS Italian patient. *Amyotroph Lateral Scler Frontotemporal Degener* **2015**, *16*, 530–531.

(20) Gibson, S. B.; Downie, J. M.; Tsetsou, S.; Feusier, J. E.; Figueroa, K. P.; Bromberg, M. B.; Jorde, L. B.; Pulst, S. M. The evolving genetic risk for sporadic ALS. *Neurology* **2017**, *89*, 226–233.

(21) Leblond, C. S.; Gan-Or, Z.; Spiegelman, D.; Laurent, S. B.; Szuto, A.; Hodgkinson, A.; Dionne-Laporte, A.; Provencher, P.; de Carvalho, M.; Orrù, S.; Brunet, D.; Bouchard, J.-P.; Awadalla, P.; Dupré, N.; Dion, P. A.; Rouleau, G. A. Replication study of MATR3 in familial and sporadic amyotrophic lateral sclerosis. *Neurobiol. Aging* **2016**, *37*, 209.e17–209.e21.

(22) Xu, Z.; Henderson, R. D.; David, M.; McCombe, P. A. Neurofilaments as Biomarkers for Amyotrophic Lateral Sclerosis: A Systematic Review and Meta-Analysis. *PLOS ONE* **2016**, *11*, 1–18.

(23) Narain, P.; Pandey, A.; Gupta, S.; Gomes, J.; Bhatia, R.; Vivekanandan, P. Targeted

next-generation sequencing reveals novel and rare variants in Indian patients with amyotrophic lateral sclerosis. *Neurobiol. Aging* **2018**, *71*, 265.e9–265.e14.

(24) Morchikh, M.; Cribier, A.; Raffel, R.; Amraoui, S.; Cau, J.; Severac, D.; Dubois, E.; Schwartz, O.; Bennasser, Y.; Benkirane, M. HEXIM1 and NEAT1 Long Non-coding RNA Form a Multi-subunit Complex that Regulates DNA-Mediated Innate Immune Response. *Mol. Cell* **2017**, *67*, 387–399.e5.

(25) Valencia, C. A.; Ju, W.; Liu, R. Matrin 3 is a Ca2+/calmodulin-binding protein cleaved by caspases. *Biochem. Biophys. Res. Commun.* **2007**, *361*, 281–286.

(26) Przygodzka, P.; Boncela, J.; Cierniewski, C. S. Matrin 3 as a key regulator of endothelial cell survival. *Exp. Cell. Res.* **2011**, *317*, 802–811.

(27) Coelho, M. B.; Attig, J.; Ule, J.; Smith, C. W. Matrin3: connecting gene expression with the nuclear matrix. *WIREs RNA* **2016**, *7*, 303–315.

(28) Yedavalli, V. S.; Jeang, K.-T. Matrin 3 is a co-factor for HIV-1 Rev in regulating post-transcriptional viral gene expression. *Retrovirology* **2011**, *8*.

(29) Sarracino, A.; Gharu, L.; Kula, A.; Pasternak, A. O.; Avettand-Fenoel, V.; Rouzioux, C.; Bardina, M.; Wit, S. D.; Benkirane, M.; Berkhout, B.; Lint, C. V.; Marcello, A.; Fassati, A.; Goff, S. P. Posttranscriptional Regulation of HIV-1 Gene Expression during Replication and Reactivation from Latency by Nuclear Matrix Protein MATR3. *mBio* **2018**, *9*, e02158–18.

(30) Salton, M.; Lerenthal, Y.; Wang, S.-Y.; Chen, D. J.; Shiloh, Y. Involvement of Matrin 3 and SFPQ/NONO in the DNA damage response. *Cell Cycle* **2010**, *9*, 1568–1576.

(31) Niimori-Kita, K.; Tamamaki, N.; Koizumi, D.; Niimori, D. Matrin-3 is essential for fibroblast growth factor 2-dependent maintenance of neural stem cells. *Sci. Rep.* **2018**, *8*.

(32) Subbarayalu, P.; Rajamanickam, S.; Viswanadhapalli, S.; Dybdal-Hargreaves, N.; Timilsina, S.; Bansal, S.; Bansal, H.; Mohammad, T.; Chen, Y.; Herr, J. C.; Mooberry, S. L.; Rao, M. K. Abstract P4-05-09: Matrin 3: A novel micro-tubule associated RNA binding protein that acts as a potent tumor suppressor. *Cancer Res.* **2015**, *75*, P4–05–09–P4–05–09.

(33) Gallego-Iradi, M. C.; Strunk, H.; Crown, A. M.; Davila, R.; Brown, H.; Rodriguez-Lebron, E.; Borchelt, D. R. N-terminal sequences in matrin 3 mediate phase separation into droplet-like structures that recruit TDP43 variants lacking RNA binding elements. *Lab. Invest.* **2019**, *99*, 1030—-1040.

(34) Salton, M.; Elkon, R.; Borodina, T.; Davydov, A.; Yaspo, M.-L.; Halperin, E.; Shiloh, Y. Matrin 3 Binds and Stabilizes mRNA. *PLOS ONE* **2011**, *6*, 1–7.

(35) Erazo, A.; Goff, S. P. Nuclear matrix protein Matrin 3 is a regulator of ZAP-mediated retroviral restriction. *Retrovirology* **2015**, *12*.

(36) Zhao, M.; Kim, J. R.; van Bruggen, R.; Park, J. RNA-Binding Proteins in Amyotrophic Lateral Sclerosis. *Mol. Cells* **2018**, *41*, 818–829.

(37) Zeitz, M. J.; Malyavantham, K. S.; Seifert, B.; Berezney, R. Matrin 3: Chromosomal distribution and protein interactions. *J. Cell. Biochem.* **2009**, *108*, 125–133.

(38) Coelho, M. B.; Attig, J.; Bellora, N.; König, J.; Hallegger, M.; Kayikci, M.; Eyras, E.; Ule, J.; Smith, C. W. Nuclear matrix protein Matrin3 regulates alternative splicing and forms overlapping regulatory networks with PTB. *EMBO J.* **2015**, *34*, 653–668.

(39) Boehringer, R.; Polygalov, D.; Huang, A. J.; Middleton, S. J.; Robert, V.; Wintzer, M. E.; Piskorowski, R. A.; Chevaleyre, V.; McHugh, T. J. Chronic Loss of CA2 Transmission Leads to Hippocampal Hyperexcitability. *Neuron* **2017**, *94*, 642–655.e9.

(40) Zhang, Z.; Carmichael, G. G. The Fate of dsRNA in the Nucleus: A p54nrb-Containing Complex Mediates the Nuclear Retention of Promiscuously A-to-I Edited RNAs. *Cell* **2001**, *106*, 465–476.

(41) Kula, A.; Guerra, J.; Knezevich, A.; Kleva, D.; Myers, M. P.; Marcello, A. Characterization of the HIV-1 RNA associated proteome identifies Matrin 3 as a nuclear cofactor of Rev function. *Retrovirology* **2011**, *8*.

(42) Hornbeck, P. V.; Zhang, B.; Murray, B.; Kornhauser, J. M.; Latham, V.; Skrzypek, E. PhosphoSitePlus, 2014: mutations, PTMs and recalibrations. *Nucleic Acids Res.* **2014**, *43*, D512–D520.
